# Impact of Halogen Substituents on Interactions between 2-Phenyl-2,3-dihydroqulinazolin-4(1*H*)-one Derivatives and Human Serum Albumin

**DOI:** 10.3390/molecules17022000

**Published:** 2012-02-17

**Authors:** Feng Liu, Yi Wang, Cong Lv, Lei Wang, Junjun Ou, Min Wang, Shangzhong Liu

**Affiliations:** Department of Applied Chemistry, China Agricultural University, Beijing 100193, China

**Keywords:** synthesis, fluorescence spectroscopy, human serum albumin (HSA), DQL

## Abstract

A novel type of 2-(un)substituted phenyl-2,3-dihydroquinazolin-4(1*H*)-one (DQL) derivatives were designed and synthesized to study the impact of halogen substituents on interactions between DQL and human serum albumin (HSA) by comparison methodology. The interactions between DQL and HSA were studied by fluorescence spectroscopy. The intrinsic fluorescence of human serum albumin was quenched by DQL through a static quenching mechanism. Site marker competitive experiments showed that DQL bound to HSA in site II (subdomain IIIA). The binding constants, the numbers of binding sites and the thermodynamic parameters were measured too. The results indicated that the interactions were spontaneous, mainly through hydrophobic forces, and the substitution by halogen atoms in the benzene ring could increase the interactions between DQL and HSA. Furthermore, the binding affinity was enhanced gradually with the increasing of halogen atomic number.

## 1. Introduction

Human serum albumin (HSA), the most abundant protein in the circulatory system, has many physiological and pharmacological functions. It is chiefly responsible for the maintenance of blood pH and contributes to colloid osmotic blood pressure. Moreover, it provides a depot and carrier for many endogenous and exogenous ligands present in blood. It plays a prevailing role in drug pharmacokinetics and pharmacodynamics due to its capacity to bind a large variety of drugs [1,2,3]. The binding affinities of drugs to HSA can strongly affect their absorption, distribution, metabolism, excretion, and their therapeutic effects. Generally, strong binding can decrease the concentrations of free drug in plasma, while weak binding can lead to short lifetime and/or poor distribution. For example, many promising new drugs were rendered ineffective due to their unusually high binding affinity to HSA [4,5,6,7]. Therefore, it is important to investigate the interactions between new compounds and HSA in the early process of drug discovery [8,9].

Heterocyclic structures form the basis of many pharmaceutical, agrochemical and veterinary products. Quinazolines are one of the most important classes of heterocyclic compounds, and many quinazoline derivatives have been reported to possess anticancer, antitubercular, antibacterial, antifungal, anti-HIV, analgesic, anti-inflammatory, anticonvulsant, antiparkinson, anti-histaminic, anthelmintic, and antihypertensive activities [10,11,12,13,14,15,16,17,18]. Thus, considerable attention has been focused by biologists and chemists on their structures and bioactivity. However, scarce information is available on the interactions of this class of compounds with serum albumin; especially, no information is available on the impact of halogen substituents on the interactions between quinazoline derivatives and serum albumin.

**Scheme 1:**
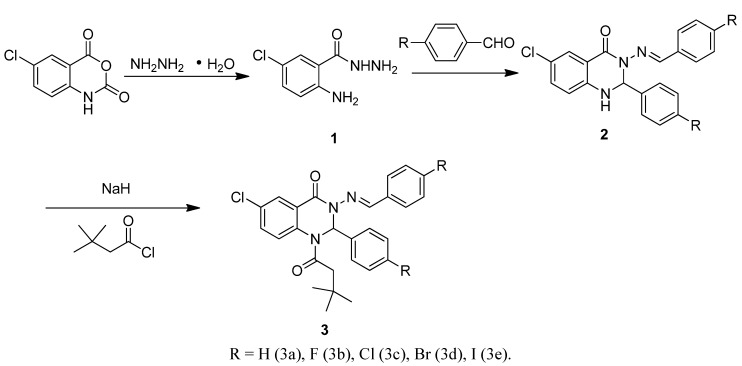
Synthetic route to the title compounds.

In this paper, we designed and synthesized a new type of 2-(un)substituted phenyl-2,3-dihydroquinazolin-4(1*H*)-one (DQL) derivatives using the synthetic route shown in Scheme 1, and investigated the binding interactions between five DQLs and HSA at three temperatures by fluorescence spectroscopy. The binding mechanisms and the binding parameters of five DQL with HSA such as binding constants, binding sites, and binding modes have been discussed. The influence of halogen substituent on DQL-HSA interactions was studied under simulated physiological conditions using 2-phenyl-2,3-dihydroquinazolin-4(1*H*)-one as reference compound. The results provide a quantitative understanding of halogen substituent effects on DQL-HSA interactions to some extent, which could be useful for further design of potential biologically active substituted quinazolinone derivatives.

## 2. Results and Discussion

### 2.1. Synthesis

A number of quinazoline derivatives were designed and synthesized in our laboratory for discovering new biological molecules based upon on this versatile skeleton, and the introduction of *t*-butylacetyl moieties on quinazolines was found to improve their binding to HSA to some extent through hydrophobic action. Thus, we designed and synthesized compounds **3a–e** to study the impact of halogen substituents on the interactions of HSA and quinazolines.

### 2.2. Fluorescence Quenching Mechanism

A variety of molecular interactions can result in quenching of compound fluorescence. These include ground-state complex formation, collisional quenching, excited state reactions, molecular rearrangement, and energy transfer with two classes of dynamic and static quenching. Dynamic and static quenching can be distinguished by their different dependence on temperature and viscosity, or preferably by lifetime measurements [19]. To confirm the mechanism of quenching fluorescence of HSA by DQL, the Stern-Volmer equations (1) and (2) [20] were used for static and dynamic quenching to analyze obtained data:

*F*_0_/*F* = 1 + *K*_SV_ [Q] (1)

**Figure 1:**
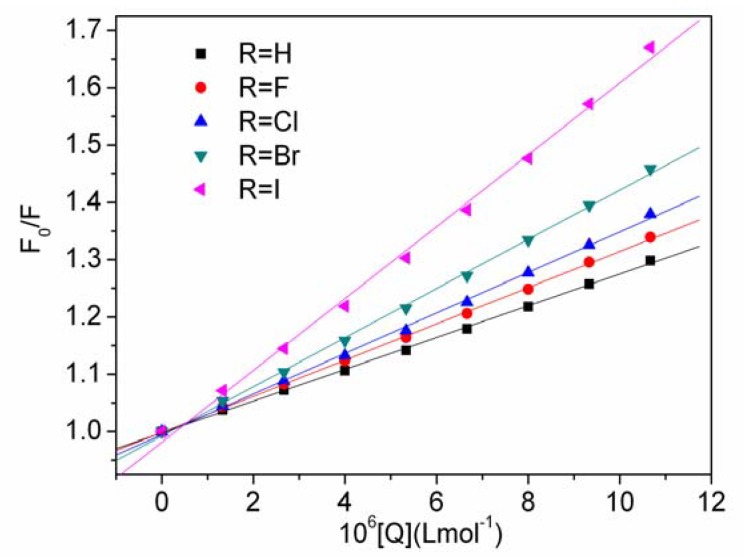
Stern-Volmer plots for DQL with HSA at 298 K.

*F*_0_/*F* = 1 + *K*q τ_0_ [Q] (2)

where *F*_0_ and *F* are the fluorescence intensities of HSA in the absence and presence of the quencher, respectively, *K*_SV_ is the Stern-Volmer quenching constant, *K*_q_ is the bimolecular quenching constant, *τ*_0_ is the average lifetime of the biomolecule without the quencher (*τ*_0_ = 10^−8^ S^−1^) [21], and [Q] is the concentration of the quencher. Figure 1 displays the Stern-Volmer plots at 298 K and different quencher concentrations. All Stern-Volmer plot curves were found to be linear with high values, and the calculated *K*_SV_ and *Kq* at the corresponding temperatures were listed in Table 1.

**Table 1:** Stern-Volmer quenching constants for the interactions of DQL with HSA at different temperatures.

Compound	T (K)	*K*_SV_(×10^4^ M^−1^)	*K*q(×10^12^ M^−1^ s^−1^)	*R* ^a^	*SD* ^b^
**R=H**	298	2.77	2.77	0.99956	0.00322
	307	2.98	2.98	0.99933	0.00424
	316	3.23	3.23	0.99919	0.00513
	298	3.14	3.14	0.99975	0.00274
**R=F**	307	3.41	3.41	0.99955	0.00398
	316	3.52	3.52	0.99925	0.00534
	298	3.54	3.54	0.99932	0.00510
**R=Cl**	307	3.64	3.64	0.99952	0.00442
	316	3.82	3.82	0.99899	0.00672
	298	4.29	4.29	0.99934	0.00610
**R=Br**	307	4.41	4.41	0.99905	0.00751
	316	4.47	4.47	0.99923	0.00686
	298	6.27	6.27	0.99837	0.01397
**R=I**	307	6.32	6.32	0.99771	0.01675
	316	6.62	6.62	0.99809	0.01600

^a^ *R* is the correlation coefficient. ^b^ *SD* is the standard deviation for the *K*_SV_ values.

It is known that linear Stern-Volmer plots indicate only one type of quenching mechanism, either static or dynamic, being predominant [22]. The plots showed that *K*_SV_ and *K*q increased as the temperature increased, which indicated that the mechanism of the quenching may be a dynamic quenching. However, the values of *K*q for all reactions of DQL-HSA in Table 1 fell in the range of 2.77 × 10^12^ to 6.62 × 10^12^ mol·L^−1^, and were much higher than 2.0 × 10^10^ mol·L^−1^, the maximum scatter collision quenching constant of various quenchers with biopolymer [23]. Hence, it could be proposed that the quenching mechanism was not initiated by dynamic collision but rather originated from the formation of a complex through a static quenching procedure.

**Figure 2:**
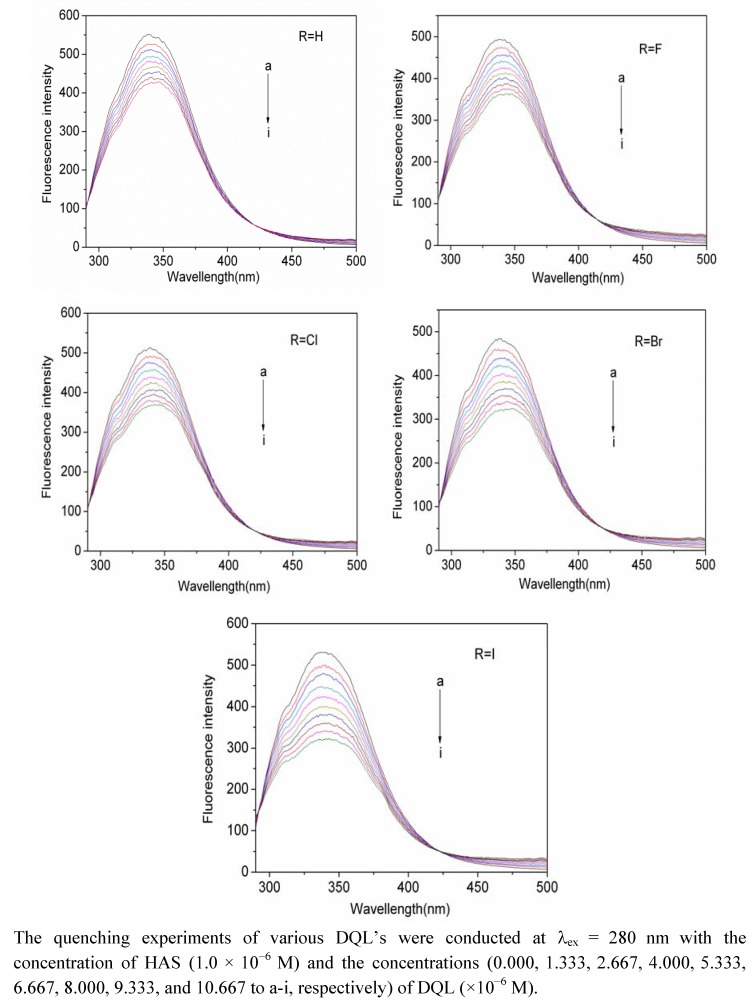
The fluorescence quenching spectra of HSA at different concentrations of DQL at 298K.

Furthermore, the effect of DQL on the fluorescence emission spectra of HSA in Figure 2 showed that the fluorescence intensity of HSA consistently decreased in the presence of increasing concentration of DQL, and a slight red shift at the maximum emission wavelength of HSA was observed. The latter indicated again that the complex between DQL and HSA formed [24]. Therefore, we conclude here the responsibility of static quenching mechanism as the dominant mechanism of the HSA fluorescence quenching by DQL.

**Figure 3:**
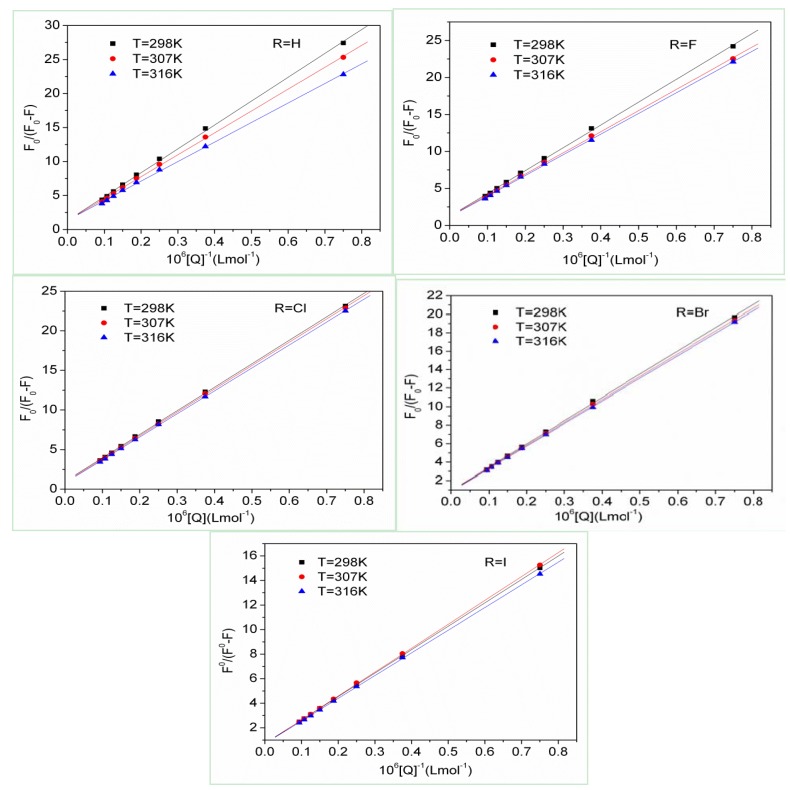
Modified Stern-Volmer plots of DQL-HSA systems.

For a complex formation process, a modified Stern-Volmer Equation (3) was used to calculate the affinity constant Ka of the binding between DQL and HSA [25,26]:

*F*_0_/(*F*_0_ − *F*) = *f*_a_^−1^·*K*_a_^−1^·[Q]^−1^ + *f*_a_^−1^ (3)

where *f*_a_ represents the fraction of accessible fluorescence and *K*_a_ is the effective quenching constant. *F*_0_/(*F*_0_ − *F*) is linear with the reciprocal value of the quencher concentration [Q], and the slope equals the value of fa−1·*K*_a_^−1^. Data were treated according to the modified Stern-Volmer equation to obtain the linear plots at different temperatures shown in Figure 3. The corresponding values of *K*_a_ in Table 2 showed that the affinity constants all increased after substitution by halogen atoms in benzene ring.

### 2.3. Binding Sites and Identification of Binding Sites on HSA

For a complex formation process, the number of binding sites can be determined according to the double-logarithmic equation [27,28]:

lg [(*F*_0_ − *F*)/*F*] = lg*K*_b_ + *n*lg[Q] (4)

where *F*_0_ and *F* are the fluorescence intensities in the absence and presence of the ligand, and *K*_b_ and n are the binding constant and the number of binding sites, respectively. According to equation (4), a plot of log[(*F*_0_ −*F*)/*F*] versus log[Q] will produce a straight line whose slope is equal to the number of binding sites. The data shown in Table 3 demonstrated a good linear relationship and values of n approximately equal to 1, which indicated that there was only one molecule of DQL binding to one molecule of HSA.

**Table 2:** Thermodynamic parameters of DQL-HSA binding systems at different temperatures.

Compound	*T* (K)	*K_a_* (×10^4^ M^−1^)	*R* ^a^	Δ*H* (kJ mol^−1^)	Δ*G* (kJ mol^−1^)	Δ*S* (Jmol^−1^ K^−1^)
**R=H**	298	2.16	0.99949		−24.72	
	307	2.34	0.99969	7.14	−25.68	106.92
	316	2.54	0.99963		−26.65	
	298	2.64	0.99967	7.60	−25.22	110.13
**R=F**	307	2.86	0.99972		−26.21	
	316	3.09	0.99981		−27.20	
	298	3.44	0.99982		−25.99	
**R=Cl**	307	3.92	0.99994	10.32	−27.08	121.84
	316	4.36	0.99995		−28.18	
	298	4.37	0.99973		−26.47	
**R=Br**	307	4.63	0.99990	5.07	−27.32	105.83
	316	4.91	0.99997		−28.37	
	298	7.21	0.99997		−26.37	
**R=I**	307	7.45	0.99993	2.80	−27.29	102.40
	316	7.69	0.99995		−28.21	

^a^ R is the correlation coefficient.

**Table 3:** Binding numbers of DQL to HSA at 298K.

compound	n	*R* ^a^	*SD* ^b^
**R=H**	0.99314	0.99945	0.01088
**R=F**	0.99313	0.99970	0.00806
**R=Cl**	1.02389	0.99956	0.00998
**R=Br**	1.03725	0.99959	0.00981
**R=I**	1.07749	0.99953	0.01087

^a^ *R* is the correlation coefficient. ^b^ *SD* is the standard deviation for the *n* values.

HSA is a globular protein composed of three homologous α-helical domains (I-III), and each domain contains two subdomains (A and B). The principal regions of ligand binding sites on albumin locate in hydrophobic cavities in subdomains IIA and IIIA [28]. For instance, phenylbutazone (PB), flufenamic acid (FA) and digitoxin (Dig) bind to site I (subdomain IIA), site II (subdomain IIIA), and site III [30,31], respectively. So, they were used as the site markers in competitive experiments to identify the binding sites of DQL on HSA. The fluorescence quenching data with the presence of site markers were analyzed using the modified Stern-Volmer Equation, and the values of binding constants listed in Table 4 showed a remarkably decrease after the addition of FA, but relatively small changes after the addition of PB and Dig. Therefore, DQL mainly bound to HSA in site II (subdomain IIIA).

**Table 4:** Effects of the site probe on the binding constants of DQL to HSA.

Compound	Site marker	*K_a_* (×10^4^ M^−1^)	*R* ^a^		*SD* ^b^
**R=H**	Blank	2.16	0.99949		0.26651
	PB	1.88	0.99962		0.27020
	FA	1.04	0.99948		0.25962
	Dig	2.18	0.99938		0.24163
**R=F**	Blank	2.64	0.99967	0.26883	
	PB	1.91	0.99816	0.49686	
	FA	1.25	0.99958	0.26440	
	Dig	2.58	0.99900	0.29079	
**R=Cl**	Blank	3.44	0.99982	0.19463	
	PB	2.88	0.99975	0.18165	
	FA	1.11	0.99986	0.19765	
	Dig	3.34	0.99974	0.15826	
**R=Br**	Blank	4.37	0.99973	0.13913	
	PB	4.17	0.99991	0.09116	
	FA	3.56	0.99993	0.08566	
	Dig	4.33	0.99958	0.15985	
**R=I**	Blank	7.21	0.99997	0.04105	
	PB	6.99	0.99995	0.05614	
	FA	4.62	0.99969	0.15900	
	Dig	7.28	0.99996	0.04216	

^a^ *R* is the correlation coefficient. ^b^ *SD* is the standard deviation for the *K_a_* values.

### 2.4. Thermodynamic Parameters and Binding Modes

The interaction forces between ligands and biomolecules include probably electrostatic interactions, multiple hydrogen bonds, van der Waals force, hydrophobic and steric contacts, and so on [32]. Generally, the signs and magnitudes of the thermodynamic parameters enthalpy change (Δ*H*) and entropy change (Δ*S*) can account for the main forces involved in the binding process. Ross and Subramanian have summarized the thermodynamic law of judging the primary binding driving force of biomacromolecules with drugs as follows: (1) Δ*H* > 0 and Δ *S* > 0 indicate a hydrophobic interaction; (2) Δ*H* < 0 and Δ*S* < 0 suggest that hydrogen bond and van der Waals force are the dominating force; (3) Δ*H * ≌ 0 and Δ*S* > 0 imply that electrostatic interactions are dominant [33].

If the enthalpy change (Δ*H*) does not vary significantly in the temperature studied, both the enthalpy change (Δ*H*) and the entropy change (Δ*S*) can be evaluated from the van’t Hoff equation:

ln*K*_a_ = −Δ*H*/*RT* + Δ*S/R* (5)

where *K*_a_ is analogous to the associative binding constants at the corresponding temperature and *R* is the gas constant.

**Figure 4:**
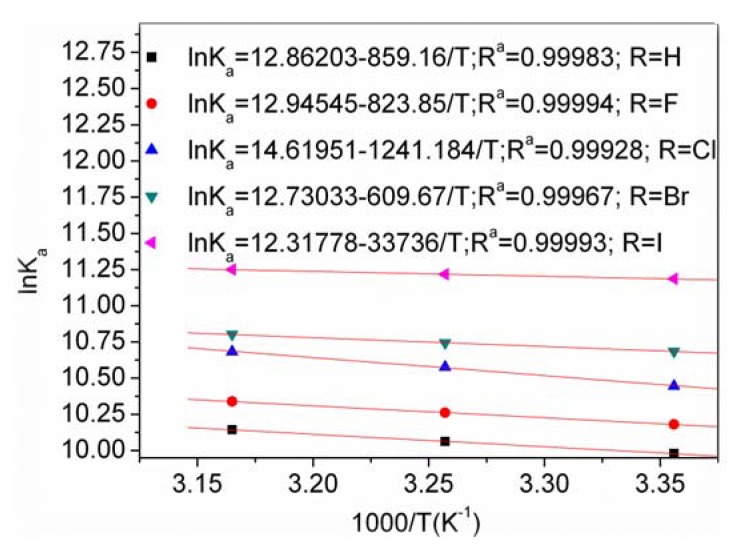
van’t Hoff plots for DQL-HSA systems.

To elucidate the interaction between DQL and HSA, the thermodynamic parameters were calculated from the van’t Hoff plots (Figure 4). The enthalpy change (Δ*H*) was calculated from the slope of the van’t Hoff relationship. The free energy change (Δ*G*) was then estimated from the following equation:

Δ*G* = Δ*H* − *T*Δ*S* (6)

As shown in Table 2, the negative signs for free energy (Δ*G*) of the DQL-HSA systems indicated that the interaction processes were spontaneous. The signs for Δ*H* and Δ*S* of the binding reaction were both found to be positive, which indicated that the binding was mainly entropy-derived and the enthalpy was unfavorable for it. Thus, the hydrophobic forces played a major role in the binding process of DQL to HSA.

With the use of 2-phenyl-2,3-dihydroquinazolin-4(1*H*)-one as reference compound, the changes of Δ*H* and Δ*S* were compared and studied after incorporating halogen atoms in benzene ring. The signs of ΔΔ*H* and ΔΔ*S* listed in Table 5 showed that the binding affinity was enhanced by hydrophobic interaction after incorporating fluorine and chlorine atoms, but by van der Waals force after incorporating bromine and iodine atoms upon on the thermodynamic law summarized by Ross and Subramanian.

**Table 5:** The values of ΔΔ*H* and ΔΔ*S.*

Compound	ΔΔ*H* (kJ mol^−1^)	ΔΔ*S* (J mol^−1^ K^−1^)
**R=F**	0.46	3.21
**R=Cl**	3.18	14.92
**R=Br**	−2.07	−1.09
**R=I**	−4.34	−4.52

In addition, the data at the corresponding temperature in Table 6 displayed the Δ*G* changes of interactions of DQL-HSA after incorporating halogen atoms in benzene ring. The values showed that the ΔΔ*G* changed only slightly as the temperature changed, and the negative sign of ΔΔ*G* indicated that incorporation of halogen atoms increased the binding affinity in the DQL-HSA systems. Furthermore, it was interestingly found that the data of ΔΔ*G* decreased regularly with the increasing halogen atomic number.

**Table 6:** The values of ΔΔ*G* at the corresponding temperature.

	*T*(K)	R=F	R=Cl	R=Br	R=I
****ΔΔ*G* (kJ mol^−1^)****	298	−0.50	−1.27	−1.75	−3.00
	307	−0.53	−1.40	−1.74	−2.96
	316	−0.55	−1.53	−1.72	−2.91

Moreover, the influence of halogen substituents on drug-protein interaction includes mainly electrostatic, steric and hydrophobic effects. The common parameters of halogen atoms are listed in Table 7 [33]. It can be seen that the atomic radius and hydrophobic parameters increase with the increasing of atomic number of the halogen, while the electronegativity decreases. According to the values of ΔΔ*G* listed in Table 6, it can be proposed that the steric and hydrophobic effects of halogen atoms played a key role in enhancing the binding affinity of the DQL-HSA systems.

**Table 7:** The common physicochemical parameters of halogens.

Element	Atomic number	Electronegativity	Atomic radius/Å	Hydrophobic parameter
**F**	9	4	0.64	0.14
**Cl**	17	3	0.99	0.71
**Br**	35	2.8	1.14	0.86
**I**	53	2.5	1.33	1.12

## 3. Experimental

### 3.1. Apparatus

Melting points were measured using a Fisher-Johns melting point apparatus (Cole-Parmer Co.) without correcting the thermometer. The NMR spectra were recorded on a Bruker Advance DPX 300 (Bruker, Karlsruhe, Germany) spectrometer using tetramethylsilane (TMS) as internal standard. The IR spectra were recorded on a Bruker Tensor 27 (Bruker Optics GmbH, Ettlingen, Germany) in KBr pellets in the range 4,000–400 cm^−1^. Mass spectra were recorded using an HPLC-1100/TOF MS high resolution mass spectrometer (Agilent Technologies. Santa Clara, CA, USA). All fluorescence spectra were measured on a Cray Eclipse fluorescence spectrophotometer (Agilent Technologies, Santa Clara, CA, USA) equipped with a thermostat bath.

### 3.2. Reagents

HSA (≥99.9, fatty-acid free), purchased from Sigma-Aldrich (St. Louis, MO, USA), was used without further purification and its molecular weight was assumed to be 66,478. Phenybutazon (PB), fluofenamic acid (FA) and digitoxin (Dig) were of analytical grade, and purchased from the National Institute for Control of Pharmaceutical and Bioproducts (Beijing, China), and the stock solutions were prepared in absolute ethanol. All other chemicals were obtained from commercial sources. Flash column chromatography with silica gel was used to purify the crude products.

### 3.3. Fluorescence Titration Experiments

All HSA solutions were prepared in buffer solution (0.1 M Tris base and 0.1 M NaCl at pH 7.4), and the HSA stock solutions were kept in the dark at 4 °C. The solution (3.0 mL) containing 1.0 × 10^−6^ M HSA was titrated by successive additions of 8.0 × 10^−4^ M ethanol stock solution of DQL (to give a final concentration of 1.333–10.667 × 10^−6^ M). Titrations were done manually by using trace syringes, and the fluorescence intensity was measured (excitation at 280 nm and emission at 337 nm). All experiments were conducted at three temperatures (298, 307, and 316 K).

### 3.4. Site Marker Competitive Experiments

Binding location studies between DQL and HSA in the presence of three site makers (phenybutazon, fluofenamic acid, digitoxin) were measured using the fluorescence titration methods. The concentration of HSA and site makers were all stabilized at 1.0 × 10^−6^ M. The solution of DQL was then gradually added to the phenybutazon-HSA, fluofenamic acid-HSA, or digitoxin-HSA mixtures, and the fluorescence intensity was recorded (excitation at 280 nm and emission at 337 nm).

### 3.5. Synthesis *of* (un)Substituted Phenyl-2,3-dihydroquinazolin-4(1*H*)-one Derivatives ***3a–3e***

Compound **1** and compounds **2a–2e** were prepared according to reported methods [35,36]. Their properties were as follows:

*2-Amino-5-chlorobenzohydrazide* (**1**): white crystals, yield 85%; m.p. 139.6–140.5 °C; ^1^H-NMR (DMSO-*d_6_*): *δ* 4.38 (d, 2H, *J* = 10.5 Hz), 6.47 (s, 2H), 6.71 (d, 1H, *J* = 8.8 Hz), 7.15 (q, 1H, *J* = 2.5 Hz, *J* = 8.8 Hz), 7.45 (d, 1H, *J* = 2.5 Hz), 9.60 (s, 1H).

*3-(Benzylideneamino)-6-chloro-2-phenyl-2,3-dihydroquinazolin-4(1*H*)-one* (**2a**): light yellow solid, yield 75.3%; m.p. 193.5–194.5 °C; ^1^H-NMR (CDCl_3_): *δ* 4.99 (d, 1H, *J* = 2.1 Hz), 6.27 (d, 1H, *J* = 2.7 Hz), 6.64 (d, 1H, *J* = 8.6 Hz), 7.23 (d, 1H, *J* = 2.5 Hz), 7.26–7.44 (m, 8H), 7.62 (m, 2H), 7.92 (d, 1H, *J* = 2.4 Hz), 9.18 (s, 1H).

*3-(4-Fluorobenzylideneamino)-6-chloro-2-(4-fluorophenyl)-2,3-dihydroquinazolin-4(1*H*)-one* (**2b**): white solid, yield 80.3%; m.p. 217.2–218.4 °C; ^1^H-NMR (CDCl_3_): *δ* 4.95 (d, 1H, *J* = 1.8 Hz), 6.23 (d, 1H, *J* = 2.6 Hz), 6.66 (d, 1H, *J* = 8.6 Hz), 6.99–7.60 (m, 9H), 7.90 (d, 1H, *J* = 2.5Hz), 9.16 (s, 1H).

*3-(4-Chlorobenzylideneamino)-6-chloro-2-(4-chlorophenyl)-2,3-dihydroquinazolin-4(1*H*)-one* (**2c**): light yellow solid, yield 76.4%; m.p. 208.2–209.4 °C; ^1^H-NMR: (CDCl_3_) *δ* 4.96 (d, 1H, *J* = 2.6 Hz), 6.22 (d, 1H, *J* = 2.9 Hz), 6.67 (d, 1H, *J* = 8.6 Hz), 7.26–7.36 (m, 7H), 7.55 (m, 2H), 7.90 (d, 1H,*J* = 2.5 Hz), 9.22 (s, 1H).

*3-(4-Bromobenzylideneamino)-6-chloro-2-(4-bromophenyl)-2,3-dihydroquinazolin-4(1*H*)-one* (**2d**): light yellow solid, yield 72.5%; m.p. 205.6–206.9 °C; ^1^H-NMR: (CDCl_3_) *δ* 4.96 (s, 1H), 6.20 (d, 1H, *J* = 2.3 Hz), 6.67 (d, 1H, *J* = 8.6 Hz), 7.26–7.52 (m, 9H), 7.89 (d, 1H,*J* = 2.4 Hz), 9.23 (s, 1H).

*3-(4-Iodobenzylideneamino)-6-chloro-2-(4-iodophenyl)-2,3-dihydroquinazolin-4(1*H*)-one* (**2e**): yellow solid, yield 67.5%; m.p. 191.6–192.9 °C; ^1^H-NMR: (CDCl_3_) *δ* 5.02 (d, 1H, *J* = 2.8 Hz), 6.18 (d, 1H, *J* = 2.9 Hz), 6.66 (d, 1H, *J* = 8.6 Hz), 7.14–7.69 (m, 9H), 7.88 (d, 1H, *J* = 2.5 Hz), 9.18 (s, 1H).

For the preparation of compounds **3a–e**, the corresponding compound **2** (3 mmol) in anhydrous tetrahydrofuran (30 mL) was cooled to 0 °C with an ice bath, and sodium hydride (3.6 mmol) was added. The mixture was stirred for 0.5 h at 0 °C followed by another 1.0 h at room temperature. *tert*-Butylacetyl chloride (3.6 mmol) in anhydrous tetrahydrofuran (5 mL) was added slowly at 0 °C in 0.5 h, and then the mixture was stirred again at room temperature overnight. The solvent was removed under vacuum, and the residue was then purified by flash chromatography using hexane and ethyl acetate (*V*/*V* = 6:1) as the eluent to obtain the title compounds.

*3-(Benzylideneamino)-6-chloro-1-(3,3-dimethylbutanoyl)-2-phenyl-2,3-dihydroquinazolin-4(1*H*)-one* (**3a**): white solid, yield 49.5%; m.p. 149.5–150.8 °C; ^1^H-NMR: (DMSO-*d_6_*) *δ* 0.97 (s, 9H), 2.70 (s, 2H), 7.18 (s, 1H), 7.26–7.33 (m, 5H), 7.45–7.63 (m, 5H), 7.78–7.84 (m, 4H), 9.19 (s, 1H); ^13^C-NMR: (CDCl_3_) *δ* 29.82, 32.28, 45.20, 71.23, 126.03, 126.49, 127.94, 128.50, 128.50, 128.75, 130.84, 131.94, 132.60, 134.28, 135.98, 136.13, 152.11, 159.26, 171.21; IR (v cm^−1^): 2,959.59, 1,686.92, 1,663.16, 1,479.55, 1,460.12, 1,422.33, 1,367.15, 1,278.13, 1,228.41, 1,182.76, 1,147.96, 960.37, 828.46, 762.81, 747.66, 695.41, 546.30; HRMS: *m/z* calc for C_27_H_26_ClN_3_O_2_ (M+H)^+^ 460.1714, found 460.1776.

*3-(4-Fluorobenzylideneamino)-6-chloro-1-(3,3-dimethylbutanoyl)-2-(4-fluorophenyl)-2,3-dihydroquinazolin-4 (1*H*)-one* (**3b**): white solid, yield 55.6%; m.p. 117.8–118.9 °C; ^1^H-NMR: (DMSO-*d_6_*) *δ* 0.96 (s, 9H), 2.69 (s, 2H), 7.14 (t, 2H, *J* = 8.8 Hz), 7.22 (d, 1H, *J* = 5.2 Hz), 7.24 (d, 1H, *J* = 5.6 Hz), 7.32 (t, 2H, *J* = 8.8 Hz), 7.64 (d, 2H, *J* = 1.3 Hz), 7.73 (s, 1H), 7.82 (t, 2H, *J* = 1.3 Hz), 7.86–7.91 (m, 1H), 9.19 (s, 1H); ^13^C-NMR: (CDCl_3_) *δ* 29.75, 32.33, 45.22, 71.31, 115.68, 115.97, 125.91, 127.90, 128.55, 129.88, 130.43, 131.98, 132.02, 132.14, 132.78, 135.81, 152.35, 159.22, 161.04, 162.76, 164.33, 166.10, 171.25; IR (v cm^−1^): 2,955.44, 1,677.13, 1,603.96, 1,509.25, 1,486.06, 1,428.02, 1,358.71, 1,271.37, 1,232.26, 1,180.33, 1,149.41, 1,092.64, 926.36, 837.29, 793.05, 713.61, 576.78, 539.42, 502.58, 456.96; HRMS: *m/z* calc for C_27_H_24_ClF_2_N_3_O_2_ (M+H)^+^ 496.1525, found 496.1586.

*3-(4-Chlorobenzylideneamino)-6-chloro-1-(3,3-dimethylbutanoyl)-2-(4-chlorophenyl)-2,3-dihydroquinazolin-4(1*H*)-one* (**3c**): white solid, yield 57.8%; m.p. 163.4–164.3 °C; ^1^H-NMR:(DMSO-*d_6_*) *δ* 0.96 (s, 9H), 2.68 (s, 2H), 7.18 (s, 1H), 7.21 (s, 1H), 7.38 (m, 2H), 7.54 (d, 2H,*J* = 1.8Hz), 7.56 (d, 2H, *J* = 1.8Hz),7.64 (d, 1H, *J* = 1.9 Hz), 7.82 (m, 2H), 7.85 (d, 1H, *J* = 1.9Hz), 9.16 (s, 1H); ^13^C-NMR: (CDCl_3_) *δ* 29.77, 32.37, 45.22, 71.14, 125.90, 127.48, 129.05, 132.28, 132.74, 132.92, 134.77, 135.79, 136.97, 152.12, 159.32, 171.30; IR (v cm^−1^): 2,957.40, 1,668.64, 1,598.98, 1,481.55, 1,424.16, 1,360.19, 1,307.77, 1,267.14, 1,229.15, 1,174.66, 1,144.17, 1,115.53, 1,090.91, 1,014.64, 865.96, 831.56, 792.32, 703.01, 572.44; HRMS: *m/z* calc for C_27_H_24_Cl_3_N_3_O_2_ (M+H)^+^ 528.0934, found 528.0950.

*3-(4-Bromobenzylideneamino)-6-chloro-1-(3,3-dimethylbutanoyl)-2-(4-bromophenyl)-2,3-dihydroquinazolin-4(1*H*)-one* (**3d**): white solid, yield 53.8%; m.p. 186.5–187.5 °C; ^1^H-NMR: (DMSO-*d_6_*) *δ* 0.95 (s, 9H), 2.66 (s, 2H), 7.11 (s, 1H), 7.13 (s, 1H), 7.51 (d, 2H, *J* = 8.6 Hz), 7.64 (d, 2H, *J* = 1.8 Hz), 7.67 (d, 2H, *J* = 1.7 Hz), 7.69 (s, 1H), 7.76 (d, 2H, 8.6 Hz), 7.82 (d, 1H, *J* = 1.0 Hz), 9.14 (s, 1H); ^13^C-NMR: (CDCl_3_) *δ* 29.74, 32.33, 45.17, 71.16, 122.89, 125.35, 125.80, 127.74, 128.60, 129.26, 131.95, 132.25, 132.91, 133.14, 135.30, 135.74, 152.11, 159.28, 171.25; IR (v cm^−1^): 2,956.63, 1,668.10, 1,591.76, 1,482.93, 1,431.24, 1,358.21, 1,312.99, 1,269.06, 1,227.36, 1,180.66, 1,149.50, 1,069.88, 1,010.18, 883.59, 820.18, 791.31, 764.64, 717.22, 544.36, 519.33; HRMS: *m/z* calc for C_27_H_24_Br_2_Cl N_3_O_2_ (M+H)^+^ 615.9924, found 615.9990.

*3-(4-Iodobenzylideneamino)-6-chloro-1-(3,3-dimethylbutanoyl)-2-(4-iodophenyl)-2,3-dihydroquinazolin-4(1*H*)-one* (**3e**): white solid, yield 50.9%; m.p. 192.4–193.5 °C; ^1^H-NMR: (DMSO-*d_6_*) *δ* 0.95 (s, 9H), 2.67 (s, 2H), 6.95 (s, 1H), 6.98 (s, 1H), 7.60 (d, 2H, *J* = 8.5 Hz), 7.63 (d, 2H, *J* = 2.3 Hz), 7.67 (d, 2H, *J* = 8.5 Hz), 7.82 (d, 2H, *J* = 1.9 Hz), 7.84 (s, 1H), 7.87 (s, 1H), 9.08 (s, 1H); ^13^C-NMR: (CDCl_3_) *δ* 29.76, 32.36, 45.18, 71.04, 94.68, 97.52, 125.86, 127.92, 128.64, 129.32, 133.73, 135.75, 135.99, 137.93, 152.20, 159.31, 171.28; IR (v cm^−1^): 2,953.57, 1,668.98, 1,586.91, 1,482.06, 1,428.98, 1,358.31, 1,313.50, 1,268.89, 1,227.89, 1,182.93, 1,148.67, 1,058.37, 1,006.36, 883.24, 861.31, 813.70, 789.17, 764.52, 713.54, 516.49; HRMS: *m/z* calc for C_27_H_24_ClI_2_N_3_O_2_ (M+H)^+^ 711.9646, found 711.9712.

## 4. Conclusions

In this paper, the interactions of five DQLs with HSA have been studied by fluorescence spectroscopy. The experimental results suggested that DQL could bind to HSA through a static quenching procedure in the site II (subdomain IIIA). The bindings of DQL to HSA were found to be spontaneous and hydrophobicity played a major role in the interactions. The studies of the impact of halogen substituents on the DQL-HSA interactions showed that substitution by halogen atoms in the benzene ring could enhance the binding affinity through the steric and hydrophobic effects; specifically, substitution by fluorine and chlorine atoms enhanced the binding affinity by hydrophobic interaction, while substitution by bromine and iodine atoms enhanced the binding affinity by van der Waals force. Furthermore, the values of ΔΔ*G* provide a quantitative understanding of halogen substituent effect on DQL-HSA interactions. This study is expected to provide some valuable information for the further research in the rational design of this series of compounds. As the functions of the same substituent in aromatic compounds are relative consistent and stable, we believe the obtained results about halogen substituent effect will also provide some valuable information for guiding the modification of other lead compounds containing benzene rings.

## Supplementary Materials

Supplementary materials can be accessed at: http://www.mdpi.com/1420-3049/17/2/2000/s1.
