# Supplementary material for: Impact of Halogen Substituents on Interactions between 2-Phenyl-2,3-dihydroqulinazolin-4(1H)-one Derivatives and Human Serum Albumin
Source: Molecules. 2012 Feb 17;17(2):2000–14. doi: 10.3390/molecules17022000 (PMC6268991; doi:10.3390/molecules17022000)
Supplement: Supplementary file 1 [file molecules-17-02000-s001.doc]

**Supplementary data:** spectrum of NMR and IR of intermediates and target compounds.

*2-Amino-5-chlorobenzohydrazide* (**1**) ^1^H-NMR.

^1^H-NMR of **1**

*3-(Benzylideneamino)-6-chloro-2-phenyl-2,3-dihydroquinazolin-4(1H)-one*(**2a**) ^1^H-NMR.

^1^H-NMR of **2a**

*3-(4-Fluorobenzylideneamino)-6-chloro-2-(4-fluorophenyl)-2,3-dihydroquinazolin-4(1H)-one* (**2b**) ^1^H-NMR.

^1^H-NMR of **2b**

*3-(4-Chlorobenzylideneamino)-6-chloro-2-(4-chlorophenyl)-2,3-dihydroquinazolin-4(1H)-one* (**2c**) ^1^H-NMR.

^1^H-NMR of **2c**

*3-(4-Bromobenzylideneamino)-6-chloro-2-(4-bromophenyl)-2,3-dihydroquinazolin-4(1H)-one* (**2d**) ^1^H-NMR.

^1^H-NMR of **2d**

*3-(4-Iodobenzylideneamino)-6-chloro-2-(4-iodophenyl)-2,3-dihydroquinazolin-4(1H)-one* (**2e**) ^1^H-NMR.

^1^H-NMR of **2e**

*3-(Benzylideneamino)-6-chloro-1-(3,3-dimethylbutanoyl)-2-phenyl-2,3-dihydroquinazolin-4(1H)-one* (**3a**) ^1^H-NMR.

^1^H-NMR of **3a**

*3-(4-Fluorobenzylideneamino)-6-chloro-1-(3,3-dimethylbutanoyl)-2-(4-fluorophenyl)-2,3-dihydroquinazolin-4(1H)-one* (**3b**) ^1^H-NMR.

^1^H-NMR of **3b**

*3-(4-Chlorobenzylideneamino)-6-chloro-1-(3,3-dimethylbutanoyl)-2-(4-chlorophenyl)-2,3-dihydroquinazolin-4(1H)-one* (**3c**) ^1^H-NMR.

^1^H-NMR of **3c**

*3-(4-Bromobenzylideneamino)-6-chloro-1-(3,3-dimethylbutanoyl)-2-(4-bromophenyl)-2,3-dihydroquinazolin-4(1H)-one* (**3d**) ^1^H-NMR.

^1^H-NMR of **3d**

*3-(4-Iodobenzylideneamino)-6-chloro-1-(3,3-dimethylbutanoyl)-2-(4-iodophenyl)-2,3-dihydroquinazolin-4(1H)-one* (**3e**) ^1^H-NMR.

^1^H-NMR of **3e**

*3-(Benzylideneamino)-6-chloro-1-(3,3-dimethylbutanoyl)-2-phenyl-2,3-dihydroquinazolin-4(1H)-one* (**3a**) ^13^C-NMR.

^13^C-NMR of **3a**

*3-(4-Fluorobenzylideneamino)-6-chloro-1-(3,3-dimethylbutanoyl)-2-(4-fluorophenyl)-2,3-dihydroquinazolin-4(1H)-one* (**3b**) ^13^C-NMR.

^13^C-NMR of **3b**

*3-(4-Chlorobenzylideneamino)-6-chloro-1-(3,3-dimethylbutanoyl)-2-(4-chlorophenyl)-2,3-dihydroquinazolin-4(1H)-one* (**3c**) ^13^C-NMR.

^13^C-NMR of **3c**

*3-(4-Bromobenzylideneamino)-6-chloro-1-(3,3-dimethylbutanoyl)-2-(4-bromophenyl)-2,3-dihydroquinazolin-4(1H)-one* (**3d**) ^13^C-NMR.

^13^C-NMR of **3d**

*3-(4-Iodobenzylideneamino)-6-chloro-1-(3,3-dimethylbutanoyl)-2-(4-iodophenyl)-2,3-dihydroquinazolin-4(1H)-one* (**3e**) ^13^C-NMR.

^13^C-NMR of **3e**

*3-(Benzylideneamino)-6-chloro-1-(3,3-dimethylbutanoyl)-2-phenyl-2,3-dihydroquinazolin-4(1H)-one* (**3a**) IR.

*3-(4-Fluorobenzylideneamino)-6-chloro-1-(3,3-dimethylbutanoyl)-2-(4-fluorophenyl)-2,3-dihydroquinazolin-4(1H)-one* (**3b**) IR.

*3-(4-Chlorobenzylideneamino)-6-chloro-1-(3,3-dimethylbutanoyl)-2-(4-chlorophenyl)-2,3-dihydroquinazolin-4(1H)-one* (**3c**) IR.

*3-(4-Bromobenzylideneamino)-6-chloro-1-(3,3-dimethylbutanoyl)-2-(4-bromophenyl)-2,3-dihydroquinazolin-4(1H)-one* (**3d**) IR.

*3-(4-Iodobenzylideneamino)-6-chloro-1-(3,3-dimethylbutanoyl)-2-(4-iodophenyl)-2,3-dihydroquinazolin-4(1H)-one* (**3e**) IR.
